# Incidence and prognostic implications of prostate-specific antigen persistence and relapse after radical prostatectomy: population-based study

**DOI:** 10.1093/jnci/djaf012

**Published:** 2025-01-17

**Authors:** Pietro Scilipoti, Hans Garmo, Rolf Gedeborg, David Robinson, Pär Stattin, Marcus Westerberg

**Affiliations:** Department of Surgical Sciences, Uppsala University, Uppsala, Sweden; Division of Experimental Oncology/Unit of Urology, URI Institution, IRCCS Ospedale San Raffaele, Milan, Italy; Department of Surgical Sciences, Uppsala University, Uppsala, Sweden; Department of Surgical Sciences, Uppsala University, Uppsala, Sweden; Department of Urology, Ryhov Hospital, Jönköping, Sweden; Department of Surgical Sciences, Uppsala University, Uppsala, Sweden; Department of Surgical Sciences, Uppsala University, Uppsala, Sweden

## Abstract

**Background:**

There has been a wide range in incidence of prostate-specific antigen (PSA) persistence and relapse after radical prostatectomy (RP) for prostate cancer (PCa). We aimed to describe incidence and prognostic implications of PSA persistence and relapse.

**Methods:**

Register-based cohort study in Sweden of men diagnosed with PCa between 2007 and 2020 who underwent RP. Risks were estimated using competing risk cumulative incidence curves. Treatment after persistence or relapse and risk of PCa death and other causes were stratified according to persistence, European Association of Urology relapse risk groups, time to relapse, and life expectancy based on age and comorbidities.

**Results:**

Among 10 700 men, the 10-year risk of PSA persistence or relapse after RP was 34% (95% confidence interval = 32% to 35%). Within 12 months of persistence/relapse, 75% of men with persistence, high-risk relapse, or early relapse (<2 years) received treatment. The 10-year risk of PCa death ranged from 12% for men with persistence to 2% in men with low-risk relapse, whereas death from other causes ranged from 11% to 16%. Risk of PCa death was 8.5% after early relapse (<2 years) and 1.4% after late relapse (>5 years).

**Conclusions:**

This population-based study estimated that one-third of men would have PSA persistence or relapse within 10 years from RP. There was a wide range in risk of death from PCa according to cancer characteristics and time to relapse. Risk of death from other causes was substantial. These factors, along with life expectancy, should inform treatment decisions for men with persistence or relapse.

## Introduction

Radical prostatectomy (RP) is a common treatment for localized prostate cancer (PCa) worldwide, with approximately 3000 RPs performed in Sweden and 80 000-90 000 RPs performed each year in the United States.^1^^,^^2^ There is a substantial risk of prostate-specific antigen (PSA) persistence or relapse, and in previous studies there has been a large range in the proportion of relapse, from 5% to 30% for PSA persistence and 20%-50% for PSA relapse.^3-10^

Most studies on incidence and prognostic implications of PSA persistence and relapse have been based on data from tertiary referral centers of excellence, and these results are unlikely to be representative of all men in the population who undergo RP.^6-10^ Many of these studies lacked detailed data on additional treatment and life expectancy.^5^^,^^6^^,^^11^

Given the high number of men who undergo RP, the large range in incidence of PSA persistence and relapse, and the often very long disease trajectory after RP and relapse, there is a need for population-based studies that take life expectancy and risk of death from other causes into account.^3^^,^^12^

The aim of this study was to estimate the incidence and prognostic implications of PSA persistence and relapse after RP in a large population-based cohort including data on subsequent treatment, time to relapse, and life expectancy based on age and comorbidity.

## Methods

### Data sources

The National Prostate Cancer Register (NPCR) of Sweden was established in 1998 and collects data on PCa care to assess adherence to national guidelines.^13^^,^^14^ In Prostate Cancer data Base Sweden (PCBase), NPCR has been linked to other health-care registers and demographic databases, including the Patient Register, the Prescribed Drug Register, the Emigration Register, the Cause of Death Register, and the Longitudinal Integrated Database for Health Insurance and Labor Market Studies, a socioeconomic database.^15^ In PCBase Xtend (eXtended Treatments and endpoints), longitudinal data on PSA and use of androgen deprivation therapy (ADT; any use of gonadotropin-releasing hormone agonist or first-generation anti-androgen [bicalutamide]) has been extracted from health-care IT systems. In PCBase Xtend longitudinal PSA test results from 12 of 21 regions in Sweden had been collected, which accounts for 47% of the Swedish male population (Supplementary Methods).^16^

Data in the study file included Gleason score at diagnosis, details on RP, prostate volume, clinical and pathological T stage, postoperative radiotherapy, PSA at date of diagnosis and during follow-up, and initiation of ADT during follow-up. Clinical data were used to categorize men according to a modification of the National Comprehensive Cancer Network (NCCN) classification.^17^ Men with high-risk and very high-risk PCa were merged into 1 group and referred to as high-risk.

Life expectancy was calculated based on age and comorbidities at date of PSA persistence or relapse.^18^ Comorbidity was quantified by the Multidimensional Diagnosis-Based Comorbidity Index and the Drug Comorbidity Index.^19^^,^^20^ Date and cause of death were extracted from the Cause of Death Register.

### Outcomes

Prostate-specific antigen persistence was defined as a PSA value not dropping to 0.10 ng/mL or lower after RP. Relapse was defined according to the NCCN definition as 2 consecutive measurements of PSA above 0.10 ng/mL after a PSA response defined as at least 1 PSA of 0.10 ng/mL or lower after RP, in line with NCCN and Swedish guidelines.^13^^,^^17^ In a sensitivity analysis, we used a definition of 2 consecutive PSA values at or above 0.20 ng/mL. For details, see Supplementary Methods.

European Association of Urology (EAU) risk groups were used: low-risk is a Gleason score of 6-7 and PSA doubling time above 12 ng/mL/months; high-risk is a Gleason score of 8-10 or PSA doubling time at or below 12 months.

### Statistical analysis

Cumulative incidence proportions for PSA persistence and relapse were computed. We considered treatment with no prior evidence of PSA persistence or relapse and death (of any cause) as a competing event. The analysis was stratified according to NCCN PCa-risk categories. Follow-up began on the date of RP and continued until the occurrence of PSA persistence, relapse, treatment without prior persistence or relapse, date of death, date of censoring, or until December 31, 2020 (Supplementary Methods).

In the analysis of subsequent treatment, follow-up began on the date of PSA persistence or relapse and continued until the occurrence of 1 of the outcomes (treatment with radiotherapy, ADT, or radiotherapy + ADT, or death), censoring, or until December 31, 2020 (Supplementary Methods). We computed cumulative incidence proportions of the outcomes according to PSA persistence and EAU biochemical recurrence risk group according to life expectancy.^21^ We used Cox regression models to calculate univariable and multivariable hazard ratios to assess predictors of treatment initiated within 12 months of PSA persistence or relapse, adjusting for calendar period.

In the analysis of death, follow-up began on the date of PSA persistence or relapse and continued until death, date of censoring, or December 31, 2023 (Supplementary Methods). The risk of PCa death and other causes after relapse was estimated using cumulative incidence proportions stratified by PSA persistence and relapse risk group. A similar analysis was performed stratified on time from RP to relapse (<2 years, 2-5 years, and >5 years).

Cumulative incidence proportions and hazard ratios were computed with 95% confidence intervals (CIs). All analyses were performed with R, version 4.3.2.^22^ The Research Ethics authority approved of the study and waived the need for informed consent.

## Results

### Study population and baseline characteristics

After exclusion of 976 men who did not fulfill inclusion criteria or had missing data, 10 700 men remained for analysis (Figure S1). There were 2755 men (26%) with low-risk PCa, 6368 men (60%) with intermediate-risk PCa, and 1577 men (15%) with high-risk PCa at diagnosis (Table 1). Baseline characteristics of the study population were similar to those of 23 156 men who underwent RP in the other Swedish regions during the same time period (Table S1).

**Table 1.** Baseline characteristics of 10 700 men in Prostate Cancer data Base Sweden Xtend who underwent radical prostatectomy in 2007-2020 and had longitudinal data on PSA and use of androgen deprivation therapy.

	Low-risk^a^	Intermediate-risk^a^	High-risk^a^	All
No. (%)	2755 (100)	6368 (100)	1577 (100)	10 700 (100)
Year of RP				
2007-2011	729 (26)	937 (15)	285 (18)	1951 (18)
2012-2016	1036 (38)	2689 (42)	706 (45)	4431 (41)
2017-2020	990 (36)	2742 (43)	586 (37)	4318 (40)
Age at RP, years				
Median (IQR)	63 (59-68)	65 (61-69)	66 (62-70)	65 (60-69)
Civil status				
No partner	768 (28)	1920 (30)	523 (33)	3211 (30)
Partner	1987 (72)	4448 (70)	1054 (67)	7489 (70)
Level of education^b^				
Low	610 (22)	1564 (25)	422 (27)	2596 (24)
Middle	1225 (44)	2839 (45)	698 (44)	4762 (45)
High	920 (33)	1965 (31)	457 (29)	3342 (31)
Income				
Q1	318 (12)	939 (15)	298 (19)	1555 (15)
Q2	397 (14)	1194 (19)	357 (23)	1948 (18)
Q3	965 (35)	2099 (33)	474 (30)	3538 (33)
Q4	1072 (39)	2130 (33)	445 (28)	3647 (34)
Missing	3	6	3	12
PSA at diagnosis, ng/mL				
Median (IQR)	5.4 (4.2-6.9)	7.0 (4.8-10.7)	13.0 (6.4-25.0)	6.6 (4.7-10.0)
PSA density, ng/mL/cc				
Median (IQR)	0.15 (0.11-0.21)	0.19 (0.13-0.28)	0.31 (0.17-0.62)	0.18 (0.13-0.28)
Missing	100	210	69	379
Clinical local stage				
cT1	2025 (74)	3901 (61)	668 (42)	6594 (62)
cT2	730 (26)	2467 (39)	614 (39)	3811 (36)
cT3-4	− ^c^	− ^c^	295 (19)	295 (2.8)
Gleason score at diagnosis				
Gleason score 6	2755 (100)	616 (9.7)	172 (11)	3543 (33)
Gleason score 7	− ^c^	5752 (90)	559 (35)	6311 (59)
Gleason score 8-10	− ^c^		846 (54)	846 (7.9)

Abbreviations: IQR = interquartile range; PSA = prostate-specific antigen; RP = radical prostatectomy.

a Prostate cancer risk categories definitions: Low risk: cT1-cT2 tumors, PSA <10 ng/mL, and Gleason Grade Group (GGG) 1 (Gleason score ≤6); Intermediate risk: (favorable intermediate-risk) cT1-cT2 and either GGG 2 (Gleason score 3 + 4) and PSA <10 ng/mL, or GGG 1 and PSA 10-19 ng/mL OR (unfavorable intermediate-risk) cT1-cT2 and either GGG 3 (Gleason score 4 + 3) and PSA <10 ng/mL, or GGG 2 and PSA 10-19 ng/mL; High risk: (high-risk) cT3 stage or GGG 4 (Gleason score 8) or PSA 20-39 ng/mL OR (very high-risk) 2-3 of the risk factors for high-risk, or GGG 5 (Gleason score 9-10), or PSA ≥40.

b Low is less than 10 years (mandatory school), intermediate is 10-12 years (high school), high is more than 12 years of education (university).

c Not applicable.

### Cumulative incidence proportion of PSA persistence and relapse

During follow-up, 758 (7.1%) men had PSA persistence, 1455 (14%) men had a PSA relapse, and 502 (4.7%) men received treatment with no documentation of persistence or relapse. The majority of the latter (n = 445; 89%) had adverse cancer characteristics in the RP specimen (eg, pT3-4, Gleason score 8-10, pN+ and/or positive surgical margins [data not shown]).

**Figure 1.**
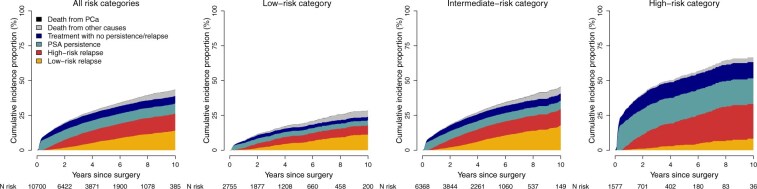
Stacked cumulative incidence proportion of PSA persistence and relapse after radical prostatectomy among 10 700 men stratified per risk category at diagnosis. Legend: only 9 men were registered as dying from prostate cancer before any of the outcomes. PSA = prostate-specific antigen.

The 10-year cumulative incidence proportion of PSA persistence was 7.2% (95% CI = 7.0% to 7.4%) and 26% (95% CI = 25% to 27%) for PSA relapse, and the cumulative incidence of persistence and relapse combined ranged from 21% to 52% depending on PCa risk category at diagnosis. At the time of PSA relapse, 836 (57%) were classified as high-risk relapse and 619 (43%) as low-risk relapse (Figure 1 and Table S2).

### Characteristics and management of men with PSA persistence or relapse

Men with PSA persistence had more adverse cancer characteristics compared with men with high-risk and low-risk relapse—for example, higher PSA at date of PSA persistence (0.40 ng/mL vs 0.20 vs 0.13 ng/mL at date of persistence vs relapse) and higher proportions of positive lymph nodes, surgical margins, and advanced local stage (Table 2).

**Table 2.** Characteristics of 758 men with PSA persistence and 1455 men with PSA relapse after radical prostatectomy stratified by relapse risk group.

	PSA persistence^a^	High-risk relapse^b^	Low-risk relapse^b^
No. (%)	758 (100)	836 (100)	619 (100)
Age at persistence or relapse, years			
Median (IQR)	66 (61-70)	68 (64-72)	69 (64-73)
Life expectancy, years			
Median (IQR)	18 (16-23)	17 (14-21)	17 (13-20)
<15	161 (21)	295 (35)	234 (38)
≥15	597 (79)	541 (65)	385 (62)
PSA at persistence/relapse, ng/mL			
Median (IQR)	0.40 (0.20-1.10)	0.20 (0.17-0.30)	0.13 (0.12-0.16)
PSA doubling time, ng/mL/months			
Median (IQR)	−^c^	7 (5-10)	21 (15-33)
Time to relapse, months			
Median (IQR)	−^c^	17 (10-33)	37 (21-56)
<24	−^c^	510 (61)	151 (25)
24-60	−^c^	266 (32)	311 (50)
>60	−^c^	60 (7.2)	157 (25)
Gleason score at RP			
Gleason score 6	137 (18)	150 (18)	177 (29)
Gleason score 7	486 (64)	529 (63)	422 (71)
Gleason score 8-10	135 (18)	157 (19)	
Pathological T stage			
T2	224 (30)	384 (46)	337 (54)
T3a	286 (38)	318 (38)	236 (38)
T3b-T4	248 (33)	134 (16)	46 (7.4)
pN+	55 (7.3)	15 (1.8)	7 (1.1)
Positive margin	440 (58)	366 (44)	225 (41)

a PSA persistence defined as a PSA value not dropping ≤0.1 ng/mL.

b Low-risk relapse is Gleason score ≤7 and PSA doubling time >12 months; high-risk relapse is Gleason score >7 or PSA doubling time ≤12 months.

c Not applicable.

Abbreviations: IQR = interquartile range; PSA = prostate-specific antigen; RP = radical prostatectomy.

**Figure 2.**
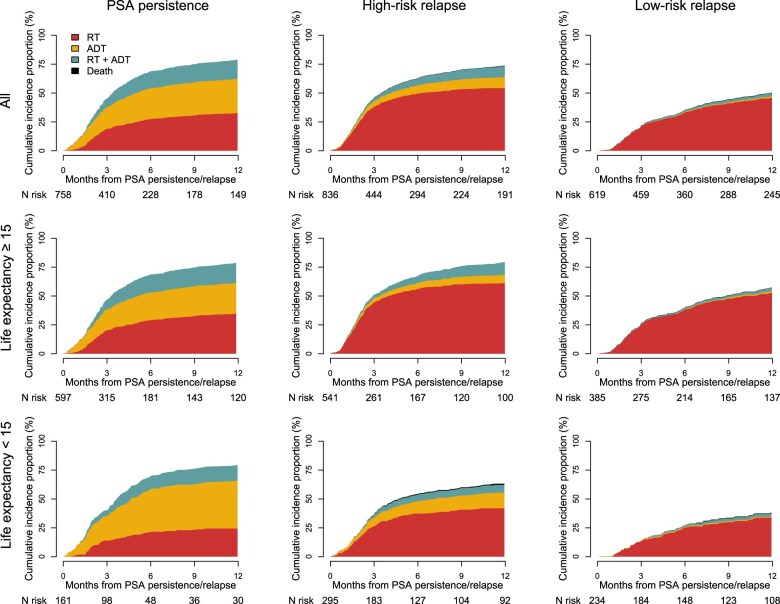
Stacked cumulative incidence proportion of treatment and death in 758 men with PSA persistence and 1455 men with PSA relapse after radical prostatectomy stratified by relapse risk groups (high-risk and low-risk) and life expectancy (computed using the comorbidities and age). PSA = prostate-specific antigen.

Combined treatment with radiotherapy and ADT or ADT only were common in men with PSA persistence with a 12-month cumulative incidence proportion of 32% and 29%, respectively, whereas radiotherapy only was the predominant treatment in men with high-risk relapse (53%) and low-risk relapse (41%) (Figure 2 and Table S3).

Treatment varied according to life expectancy, time to relapse, and region of residence (Figure 2 and Figure S2 and S3). For example, 79% of men with high-risk relapse were treated within 12 months of relapse, whereas 75% and 59% of men with life expectancy at or more than 15 years and less than 15 years, respectively, were treated (Figure 2). Most men (76%) with high-risk relapse and an early relapse (<2 years) were treated, whereas few men (37%) with late relapse (>5 years) were treated (Figure S3).

### Risk of death after PSA persistence and relapse

**Figure 3.**
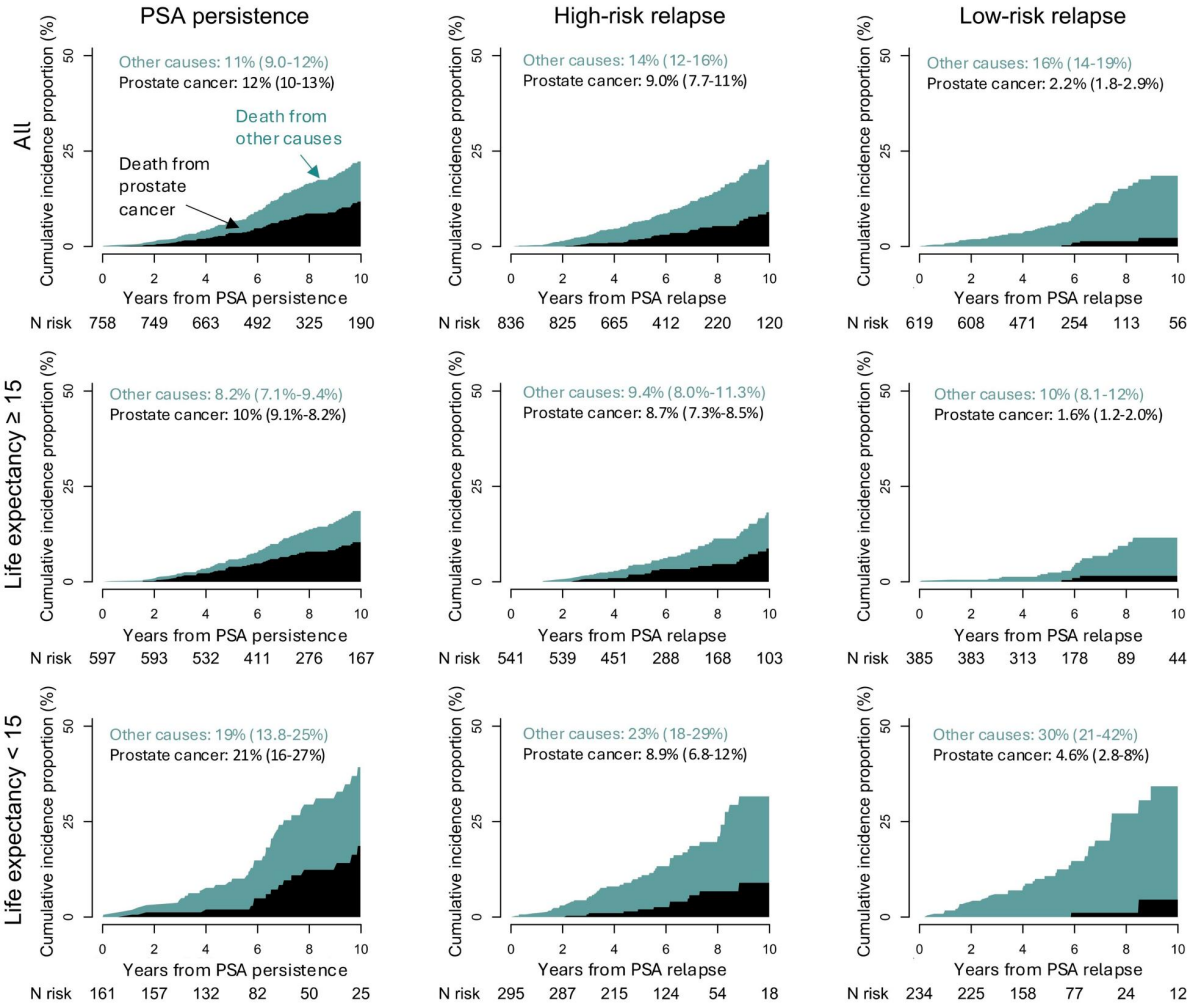
Stacked cumulative incidence proportion of death from prostate cancer and other causes in in 758 men with prostate-specific antigen (PSA) persistence and 1455 men who experienced PSA relapse after radical prostatectomy stratified by relapse risk groups (high-risk and low-risk) and life expectancy (computed from age and comorbidity).

**Figure 4.**
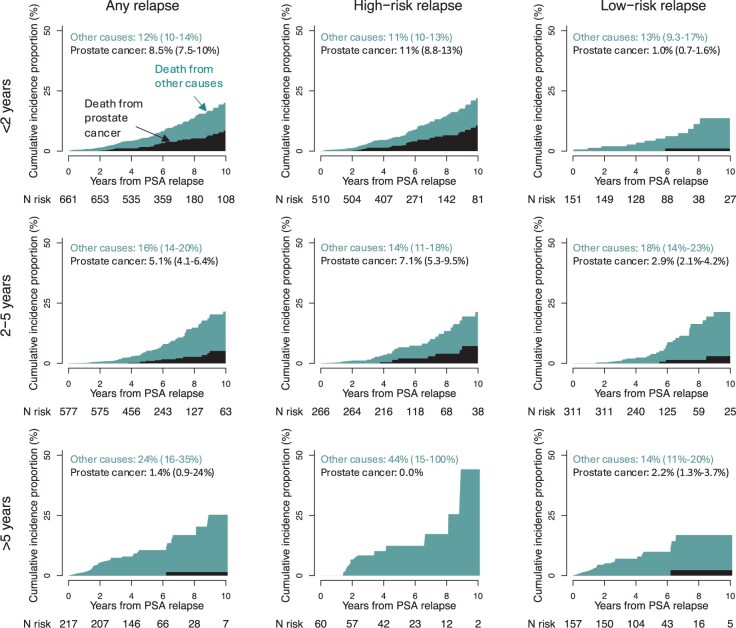
Stacked cumulative incidence proportion of death from prostate cancer and other causes in 1455 men who experienced relapse after radical prostatectomy (RP) stratified according to the time to relapse from RP. PSA = prostate-specific antigen.

The 10-year risk of PCa death ranged from 12% (95% CI = 10% to 13%) in men with PSA persistence to 2.2% (95% CI = 1.8% to 2.9%) in men with low-risk relapse, whereas the risk of death from other causes ranged from 11% (95% CI = 9.0% to 12%) to 16% (95% CI = 14% to 19%), respectively (Figure 3). In men with high-risk PSA relapse, risk of PCa death was higher (21%) after a relapse within 2 years compared with a relapse after 2-5 years (8.9%). No man in our study with a relapse more than 5 years after RP died of PCa during follow-up (Figure 4).

### Sensitivity analysis using a higher PSA cutoff

Among men who had PSA relapse based on the definition of 2 PSA values above 0.10 ng/mL, 756 (52%) also met the criteria for relapse using the stricter definition of 2 PSA values above 0.20 ng/mL. Using the 0.20 ng/mL definition, treatment patterns and the risk of PCa death after relapse were consistent with those observed using the 0.10 ng/mL definition (Figure S4).

## Discussion

### Summary of findings

In this population-based study in Sweden, one-third of men who underwent radical prostatectomy in 2007-2020 had PSA persistence or a PSA relapse during follow-up. Risk of PSA persistence and relapse was strongly associated with adverse cancer characteristics at diagnosis. Risk of PCa death was related to cancer characteristics and short time to relapse, and similar to or much lower than risk of death from other causes, which was related to life expectancy.

### Strengths and limitations

A major strength of our study is the virtually complete capture of radical prostatectomies to NPCR, which includes less than 98% of men with prostate cancer in the Cancer Register, to which reporting is mandated by law. Furthermore, the Prescribed Drug Register, the Patient Register, and the Cause of Death Register are nationwide, population-based registers with high validity.^23-26^ Access to longitudinal data on PSA and ADT during follow-up is another strength of our study. Life expectancy, which is an essential basis for treatment selection, was estimated using 2 new comorbidity indices: the Drug Comorbidity Index and the Multi-dimensional Diagnosis-based Comorbidity Index, which have both been shown to better predict death than the commonly used Charlson Comorbidity Index.^18-20^^,^^26^^,^^27^ Finally, baseline characteristics for men in our study were similar to those of men who underwent RP in other Swedish regions, supporting that our results are representative for all men who underwent RP in Sweden during this time period.

A limitation of our study is that we did not have data on imaging performed at the time of relapse (eg, prostate-specific membrane antigen positron emission tomography [PSMA PET]). Although PSMA PET was not available during most of the study period, it is likely that some men currently diagnosed with a relapse will be diagnosed with disseminated disease at an earlier time point and receive novel treatments already at that time. However, in our study, few men with low PSA levels at relapse underwent imaging, and there was a low yield of positive findings with the dominant methods in use during the study period.^13^^,^^14^ This has no impact on our estimates of PSA persistence or time to PSA relapse, but our estimates of PCa death may be slightly higher than what can be expected in men diagnosed with a PSA relapse today. There may be differences in survival according to subsequent treatments that patients received for persistent disease or after relapse. However, a comparison of outcome after different secondary and tertiary treatments requires a larger sample size, a longer follow-up, and measurement of all confounders.

### Interpretation of findings and previous studies

#### Risk of persistence and BCR

Previous observational studies have shown a wide range in the proportion of men with PSA persistence and relapse after RP, from 5% to 30% for PSA persistence^4^^,^^7^^,^^8^^,^^9^ and from 20% to 50% for PSA relapse^3^^,^^4^^,^^5^^,^^6^^,^^11^ In the SPCG-4 trial, the 10-year risk of relapse was 30% in men with Gleason score 3 + 3 (30%) and almost 90% in men with Gleason score at or above 8. We obtained similar results; risk of PSA persistence or relapse was 2.5-fold higher in men with high-risk compared with low-risk PCa.^28^

To the best of our knowledge, the only previous population-based study on PSA relapse after RP was based on data from the Stockholm region in Sweden, a region that was not included in our study. In that study, incidence of PSA persistence was not assessed.^5^ Despite different definitions of relapse (in that study 2 PSA values >0.20 ng/mL, whereas we used 0.10 ng/mL as a cutoff), the estimated risk of PSA relapse was similar, supporting that our results are representative.

#### Treatment of men with PSA persistence and PSA relapse

Treatment of PSA persistence and relapse largely aligned with recommendations in current guidelines.^13^^,^^14^ Men who had PSA persistence had a more aggressive PCa at RP and were often managed with ADT or radiotherapy plus ADT. In contrast, radiotherapy was the most common treatment for men with relapse, and this was most commonly used in men with a long life expectancy and high-risk relapse or short time to relapse.^13^^,^^14^ There were differences in use of subsequent treatments at persistence/relapse between regions, and these differences constitute a future area of analysis.

#### Prognostic implications of PSA persistence and EAU relapse risk groups

A substantial proportion of men with PSA persistence had adverse cancer characteristics, including positive surgical margins, advanced T stage, and positive lymph nodes at the time of RP. Consequently, these men had a higher risk of PCa death (12%) than men with high-risk (9.0%) or low-risk (2.2%) PSA relapse. These risk estimates are in line with the results in previous studies.^5^^,^^6^^,^^7^^,^^11^ For instance, in the previously cited Swedish study, the net risk of PCa death at 10 years was 9% for men with high-risk relapse and 4% for men with low-risk relapse.^5^

#### PSA doubling time and time to relapse

Several different algorithms are in use to calculate PSA doubling time, and they all require multiple PSA values.^29^^,^^30^ In contrast, time to relapse is easy to determine, and this was a strong predictor of risk of PCa death in our study. Men with early relapse (<2 years) had a substantially higher risk of PCa death compared with men with late relapse (>5 years), especially within the high-risk relapse group.^31^

#### Life expectancy and risk of death from other causes

Men with PSA persistence had similar risk of death of PCa and of other causes, whereas men with high-risk relapse had a 2-fold higher risk of death from other causes than from PCa and men with a low-risk relapse had an 8-fold higher risk of death from other causes. The difference varied according to life expectancy. Given the substantial risk of death from other causes and risk of adverse of treatment for both radiotherapy and ADT, treatment decisions at PSA persistence or relapse should also account for life expectancy in order to avoid overtreatment of men who have little to gain from intervention.^32^^,^^33^ Therefore, treatment at date of PSA relapse is rarely warranted in men with low-risk relapse, a relapse a long time after surgery, and/or a short life expectancy.

### Generalizability

We argue that the results on incidence and prognostic implications of PSA persistence and relapse after RP from our population-based study of men who underwent RP are likely to be representative also for other countries in which similar treatment guidelines are implemented.

### Conclusions

In conclusion, in this large population-based study it was estimated that one-third of men who underwent RP would have PSA persistence or relapse within 10 years from RP. There was a wide range in risk of PCa death after persistence or relapse according to cancer characteristics and time to relapse. Additionally, the risk of death from other causes was substantial. These factors, along with life expectancy, should inform treatment decisions for men with PSA persistence or relapse.

## Supplementary Material

djaf012_Supplementary_Data

## Data Availability

Data used in the present study were extracted from the Prostate Cancer Database Sweden (PCBase), which is based on the National Prostate Cancer Register (NPCR) of Sweden and linked to several national health-data registers. The data cannot be shared publicly because the individual-level data contain potentially identifying and sensitive patient information and cannot be published due to legislation and ethical approval (https://etikprovningsmyndigheten.se). Use of the data from national health-data registers is further restricted by the Swedish Board of Health and Welfare (https://www.socialstyrelsen.se/en/) and Statistics Sweden (https://www.scb.se/en/), which are Government Agencies providing access to the linked health-care registers. The data will be shared on reasonable request in an application made to any of the steering groups of NPCR and PCBase (contact npcr@npcr.se). To request data or analytic code from this study, contact the corresponding author. For detailed information, please see www.npcr.se/in-english, where registration forms, manuals, and annual reports from NPCR are available alongside a full list of publications from PCBase.
