# Supplementary material for: Incidence and prognostic implications of prostate-specific antigen persistence and relapse after radical prostatectomy: population-based study
Source: J Natl Cancer Inst. 2025 Jan 17;117(6):1142–50. doi: 10.1093/jnci/djaf012 (PMC12145906; doi:10.1093/jnci/djaf012)
Supplement: djaf012_Supplementary_Data [file djaf012_supplementary_data.zip › djaf012_Supplementary_Data/Supplementary figure 1.pdf]

Supplementary figure 1. Flow chart of the study population in Prostate Cancer data Base Sweden (PCaBaSe) Extend of men with prostate cancer who according to the National Prostate Cancer Register (NPCR) underwent radical prostatectomy and have complete longitudinal coverage on PSA data and androgen deprivation therapy.

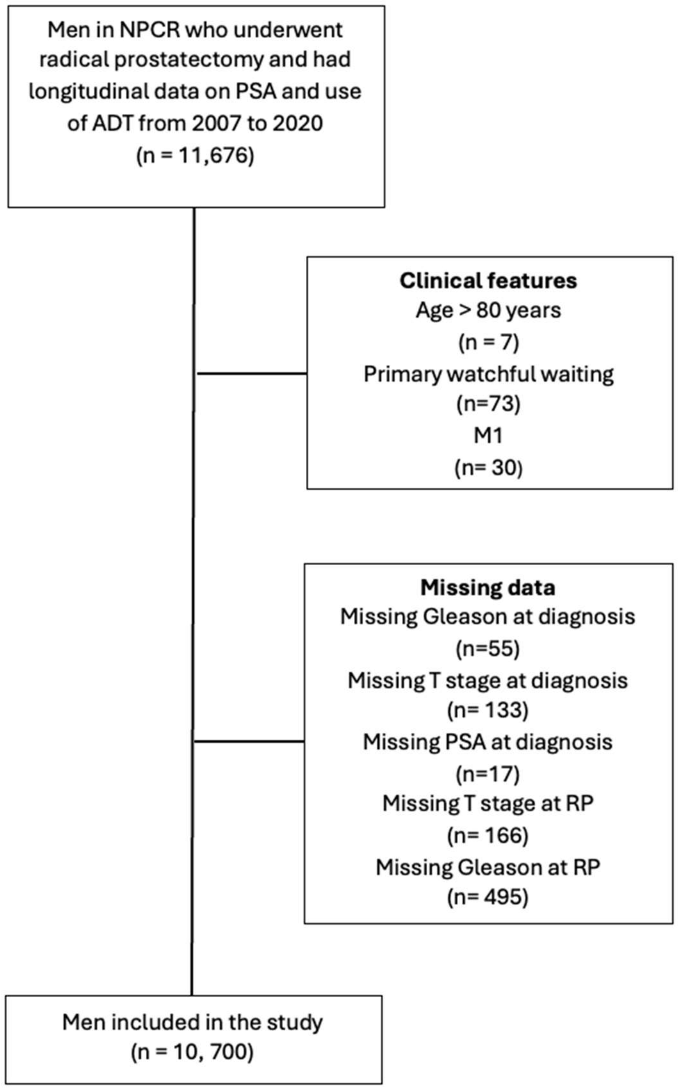

Abbreviation: NPCR, National Prostate Cancer Register; RP, radical prostatectomy, ADT androgen deprivation therapy.
