# Supplementary material for: Incidence and prognostic implications of prostate-specific antigen persistence and relapse after radical prostatectomy: population-based study
Source: J Natl Cancer Inst. 2025 Jan 17;117(6):1142–50. doi: 10.1093/jnci/djaf012 (PMC12145906; doi:10.1093/jnci/djaf012)
Supplement: djaf012_Supplementary_Data [file djaf012_supplementary_data.zip › djaf012_Supplementary_Data/Supplementary figure 2.pdf]

Supplementary figure 2. Hazard ratios (HRs) and 95% confidence intervals (CI) of any treatment within 12 months after PSA persistence/relapse.

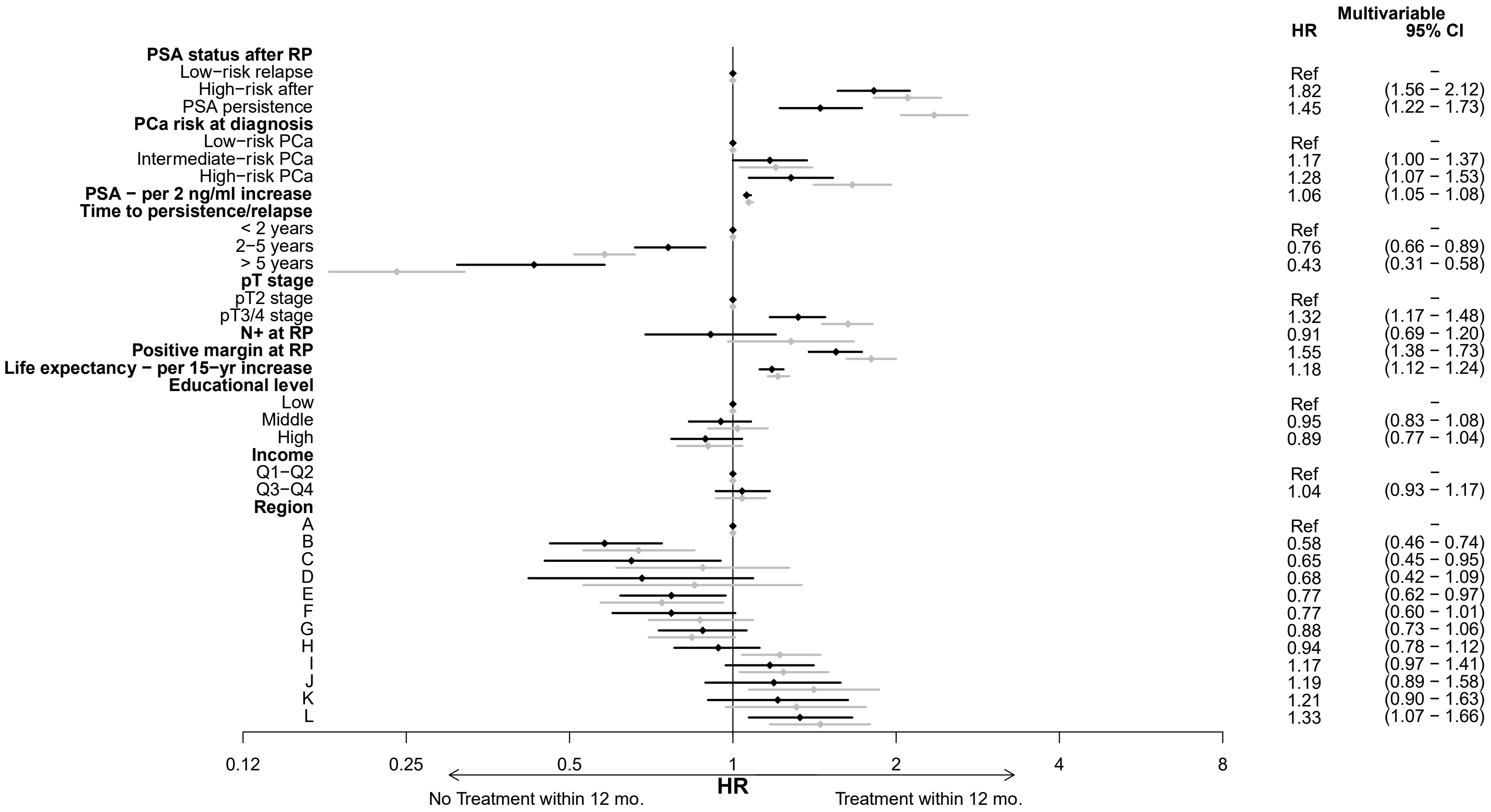

The region with the highest proportion of men with PSA persistence/relapse was used as reference. Results from univariable cox regression model are shown in grey and results from multivariable cox regression model are shown in black. For men who were not treated within 1 year after PSA persistence, the median number of PSA tests taken was 3 (IQR: 2-4), with a median maximum PSA level of 0.4 ng/mL (IQR: 0.19-0.9). Similarly, for men who were not treated within 1 year after relapse, the median number of PSA tests was also 3 (IQR: 2-4), with a median maximum PSA level of 0.2 ng/mL (IQR: 0.15-0.30).
