# Supplementary material for: Incidence and prognostic implications of prostate-specific antigen persistence and relapse after radical prostatectomy: population-based study
Source: J Natl Cancer Inst. 2025 Jan 17;117(6):1142–50. doi: 10.1093/jnci/djaf012 (PMC12145906; doi:10.1093/jnci/djaf012)

Supplementary figure 3. Cumulative incidence proportion of treatment within 12 months in 1,455 men with relapse stratified for high and low-risk relapse and time to BCR (<2 years), 2-5 years and > 5 years.

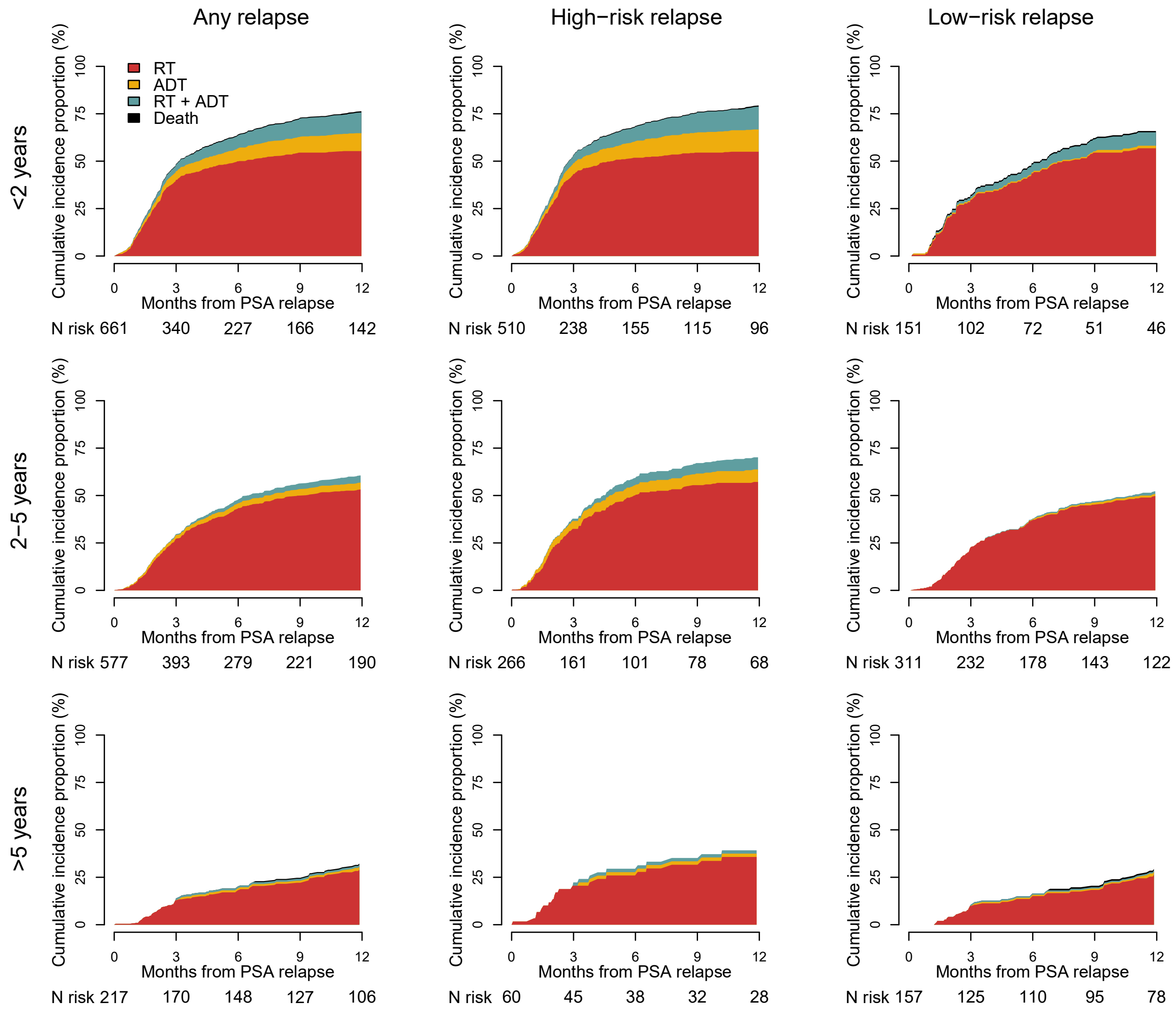

Supplement: djaf012_Supplementary_Data [file djaf012_supplementary_data.zip › djaf012_Supplementary_Data/Supplementary figure 3.pdf]
