# Supplementary material for: Incidence and prognostic implications of prostate-specific antigen persistence and relapse after radical prostatectomy: population-based study
Source: J Natl Cancer Inst. 2025 Jan 17;117(6):1142–50. doi: 10.1093/jnci/djaf012 (PMC12145906; doi:10.1093/jnci/djaf012)
Supplement: djaf012_Supplementary_Data [file djaf012_supplementary_data.zip › djaf012_Supplementary_Data/Supplementary figure 4.pdf]

Supplementary figure 4. Sensitivity analysis using a PSA cut-off of 0.20 ng/mL assessing the cumulative incidence proportion of post-RP adverse outcomes, treatment after PSA relapse and risk of death from PCa and other causes after PSA relapse.

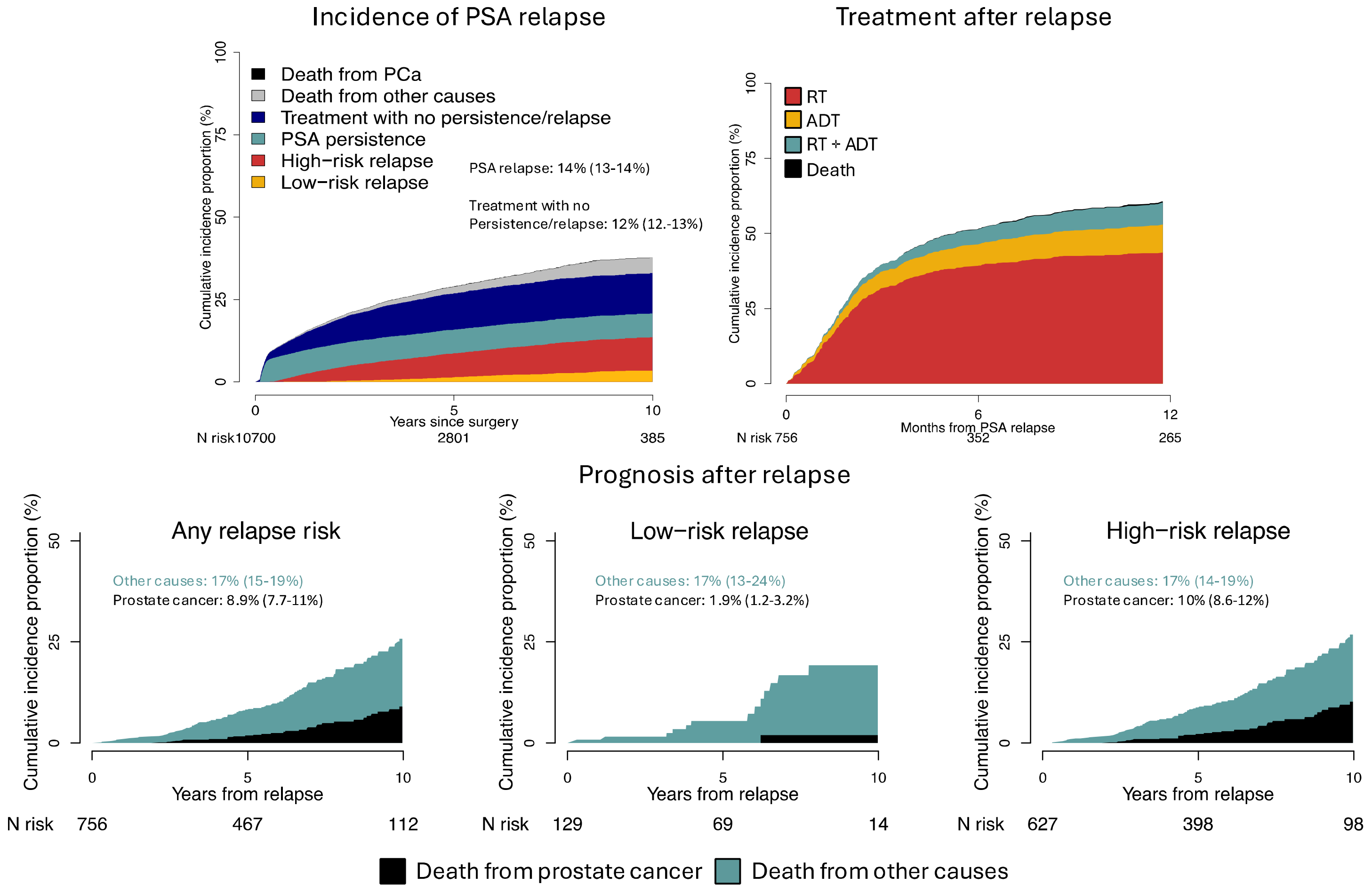

In total, 453 men (31%) received treatment after radical prostatectomy before reaching the biochemical recurrence threshold of 0.20 ng/mL but after meeting the 0.10 ng/mL criteria. For those who fulfilled both relapse definitions, the median time between reaching the 0.10 ng/mL and 0.20 ng/mL thresholds was 25 days (IQR: 0-262 days).
