# Supplementary material for: Incidence and prognostic implications of prostate-specific antigen persistence and relapse after radical prostatectomy: population-based study
Source: J Natl Cancer Inst. 2025 Jan 17;117(6):1142–50. doi: 10.1093/jnci/djaf012 (PMC12145906; doi:10.1093/jnci/djaf012)
Supplement: djaf012_Supplementary_Data [file djaf012_supplementary_data.zip › djaf012_Supplementary_Data/Supplementary methods.pdf]

Supplementary methods to “Incidence and prognostic implications of PSA persistence and relapse after radical prostatectomy. Population-based study”

Longitudinal PSA data

Additional longitudinal PSA data has been collected from regional health-care IT systems and laboratories (12 out of 21 regions) and has been integrated into PCBaSe Xtend. Current availability of PSA data varied by region as indicated in the table below.

The most common detection limit (threshold for lowest value reported) was 0.10 ng/mL but lower thresholds were occasionally used. We harmonized the data to have a uniform threshold of 0.10 ng/mL. The PSA value was occasionally reported without indication of whether the value was below or at the detection limit, so a PSA value of 0.10 ng/mL was considered as ≤ 1.0 ng/mL.

Longitudinal data on use of hormonal treatment

Information on use of hormonal treatment (gonadotropin-releasing hormone; GnRH) was retrieved from The Prescribed Drug Register. Some regions have started to administer GnRH on site and in that case, it is not registered in The Prescribed Drug Register. Longitudinal data on hospital administered drugs including GnRH has been collected from regional health-care IT systems and integrated into PCBaSe Xtend. Current availability of data on GnRH use, where data from The Prescribed Drug Register is complemented with data from regional health-care IT systems, is indicated in the table below.

**Region**	**PSA data availability**		**GnRH data availability**	
	Start date	Stop date	Start date	Stop date
Dalarna	2006-01-01	2023-04-30	2005-07-01	2022-12-31
Gävleborg	2008-01-01	2023-04-30	2005-07-01	2022-12-31
Halland	2010-01-01	2022-10-31	2005-07-01	2022-12-31
Jönköping	2011-01-01	2022-05-31	2005-07-01	2022-12-31
Kalmar	1999-10-01	2021-12-31	2005-07-01	2021-12-31
Skåne	2014-10-01	2022-10-31	2001-11-01	2022-12-31
Uppsala	2005-09-01	2021-12-31	2005-07-01	2021-12-31
Värmland	2005-01-01	2023-10-31	2005-07-01	2012-12-31
Västerbotten	1998-09-01	2023-03-31	2005-07-01	2018-12-31
Västernorrland	2014-01-01	2020-12-31	2005-07-01	2022-12-31
Örebro*	1999-10-01	2022-09-30	2005-07-01	2022-11-30
Östergötland	2000-12-01	2022-12-31	2005-07-01	2021-12-31

*Data on GnRH not available between 2014-01-01 to 2016-04-30

Definition of outcomes

A man was considered *PSA responsive* at the date when PSA first dropped to ≤0.1 within 180 days after RP, ignoring any PSA values within the first 42 days >0.10 ng/ml. *PSA persistence* was defined according to the EAU guidelines as a first PSA value >0.10 ng/ml within 180 days after RP, ignoring any PSA values within the first 42 days >0.10 ng/ml, if the man was not *PSA responsive* prior to that. *PSA relapse* was defined as two consecutive measurements of PSA >0.10 ng/ml after being *PSA responsive*. The alternative definitions for *PSA relapse* were also applied after date of being *PSA responsive*.

Censoring

In the analysis of incidence, men who emigrated or moved to a region where data on longitudinal PSA or ADT was not available were censored when they migrated/moved, and men living in regions where such longitudinal data was no longer available were censored at the time when data was not available. Men who did not take a PSA after more than two years since the most recent PSA test were censored at the 2-year mark. There were 601 men (5.6%) that were censored after moving to a region where data on PSA or ADT was not available or that did not take a PSA test for 2 years.

In the analysis of treatment, men who emigrated or moved to a region where longitudinal data on ADT was not available were censored when they migrated/moved, and men living in regions where such longitudinal data was no longer available were censored at the time when data was not available.

In the analysis of mortality, men who emigrated were censored on that date.

Computation of PSA doubling time

The PSA doubling time was calculated using the last PSA value below 0.10 ng/ml (which was set to 0.10 ng/ml since the detection limit was 0.10 ng/ml) and the subsequent two PSA values above 0.10 ng/ml following PSA responsiveness, the last of which that defined the date of PSA relapse.

To determine the doubling time, a linear regression model was applied to the natural logarithm of these three PSA values. The estimated slope of the resulting fitted line was then used to estimate the PSA doubling time using the formula log(2)/slope.
