# Supplementary material for: Incidence and prognostic implications of prostate-specific antigen persistence and relapse after radical prostatectomy: population-based study
Source: J Natl Cancer Inst. 2025 Jan 17;117(6):1142–50. doi: 10.1093/jnci/djaf012 (PMC12145906; doi:10.1093/jnci/djaf012)
Supplement: djaf012_Supplementary_Data [file djaf012_supplementary_data.zip › djaf012_Supplementary_Data/Supplementary table 1.docx]

| Characteristic | **No. (%) of men** | |
| --- | --- | --- |
|  | **Included** | **Non included** |
| No. (%) | 10,700 (100) | 23,156 (100) |
| Year of surgery |  |  |
| 2007-2011 | 1,951 (18) | 8,173 (35) |
| 2012-2016 | 4,431 (41) | 8,220 (35) |
| 2017-2020 | 4,318 (40) | 6,763 (29) |
| Age at surgery, years |  |  |
| Median [Interquartile range (IQR)] | 65 (60 - 69) | 64 (60 - 68) |
| Civil status |  |  |
| No partner | 3,211 (30) | 7,290 (31) |
| Partner | 7,489 (70) | 15,866 (69) |
| Level of education* |  |  |
| Low | 2,596 (24) | 5,222 (23) |
| Middle | 4,762 (45) | 9,761 (42) |
| High | 3,342 (31) | 8,173 (35) |
| Income |  |  |
| Q1 | 1,555 (15) | 3,102 (13) |
| Q2 | 1,948 (18) | 3,637 (16) |
| Q3 | 3,538 (33) | 6,952 (30) |
| Q4 | 3,647 (34) | 9,408 (41) |
| Missing | 12 | 57 |
| PSA at diagnosis, ng/mL |  |  |
| Median [Interquartile range (IQR)] | 6.6 (4.7 - 10.0) | 6.3 (4.5 - 9.6) |
| PSA density, ng/mL/cc |  |  |
| Median [Interquartile range (IQR)] | 0.18 (0.13 - 0.28) | 0.18 (0.13 - 0.27) |
| Missing | 379 | 1,056 |
| Clinical stage |  |  |
| cT1 | 6,594 (62) | 15,679 (68) |
| cT2 | 3,811 (36) | 6,937 (30) |
| cT3-4 | 295 (2.8) | 540 (2.3) |
| Gleason at diagnosis |  |  |
| Gleason score 6 | 3,543 (33) | 8,152 (35) |
| Gleason score 7 | 6,311 (59) | 13,398 (58) |
| Gleason score 8-10 | 846 (7.9) | 1,606 (6.9) |
| Risk category at diagnosis |  |  |
| Low risk | 2,755 (26) | 7,379 (32) |
| Intermediate risk | 6,368 (60) | 12,966 (56) |
| High risk | 1,577 (15) | 2,811 (12) |
| Pathological T stage |  |  |
| pT2 | 6,879 (64) | 15,437 (67) |
| pT3a | 2,928 (27) | 5,976 (26) |
| pT3b-T4 | 872 (8.1) | 1,662 (7.2) |
| pTx | 21 (0.2) | 81 (0.3) |
| Gleason score at RP |  |  |
| Gleason score 6 | 2,886 (27) | 8,152 (35) |
| Gleason score 7 | 6,926 (65) | 13,398 (58) |
| Gleason score 8-10 | 888 (8.3) | 1,606 (6.9) |
| Pathological N stage |  |  |
| pN0 | 3,700 (35) | 7,460 (32) |
| pN+ | 180 (1.7) | 159 (0.7) |
| pNx | 6,820 (64) | 15,537 (67) |
| Margins status |  |  |
| Negative | 6,988 (65) | 16,338 (71) |
| Positive | 3,062 (29) | 5,469 (24) |
| Unclear | 70 (0.7) | 192 (0.8) |
| Missing | 580 (5.4) | 1,157 (5.0) |

**Supplementary table 1.** Comparison of characteristics of 10,700 men who underwent radical prostatectomy and had complete PSA and GnRH data who were included in the study and 23,156 men who underwent radical prostatectomy that were not included in the study because they resided in regions where PSA and GnRH data was not available.

*Low: less than 10 years (mandatory school), intermediate: 10–12 years (high school), high: more than 12 years of education (university)
