# Supplementary material for: Incidence and prognostic implications of prostate-specific antigen persistence and relapse after radical prostatectomy: population-based study
Source: J Natl Cancer Inst. 2025 Jan 17;117(6):1142–50. doi: 10.1093/jnci/djaf012 (PMC12145906; doi:10.1093/jnci/djaf012)
Supplement: djaf012_Supplementary_Data [file djaf012_supplementary_data.zip › djaf012_Supplementary_Data/Supplementary table 2.docx]

**Supplementary table 2.** Cumulative incidence proportion and 95% confidence intervals (CI) of PSA persistence and relapse after radical prostatectomy at 10 years.

| Risk category | PSA persistence | High-risk relapse | Low-risk relapse | PSA persistence or relapse |
| --- | --- | --- | --- | --- |
| **All [95% CI]** | 7.2 (7.0-7.4) | 13 (13-14) | 12 (12-14) | 34 (32-35) |
| **Low risk prostate cancer [95%CI]** | 3.6 (3.4-3.8) | 7.8 (7.2-8.4) | 10 (9.2-11) | 21 (20-23) |
| **Intermediate risk cancer [95%CI]** | 6.0 (5.8-5.1) | 13 (13-14) | 16 (15-17) | 35 (33-38) |
| **High risk prostate cancer [95%CI]** | 18 (17-20) | 25 (23-28) | 7.8 (6.8-9) | 52 (46-58) |
