# Supplementary material for: Incidence and prognostic implications of prostate-specific antigen persistence and relapse after radical prostatectomy: population-based study
Source: J Natl Cancer Inst. 2025 Jan 17;117(6):1142–50. doi: 10.1093/jnci/djaf012 (PMC12145906; doi:10.1093/jnci/djaf012)
Supplement: djaf012_Supplementary_Data [file djaf012_supplementary_data.zip › djaf012_Supplementary_Data/Supplementary table 3.docx]

**Supplementary table 3.** Cumulative incidence proportion of salvage treatment after PSA persistence or relapse after radical prostatectomy within 12 months from PSA persistence/relapse.

|  | **PSA persistence [95% CI]** | | | **High risk relapse [95%CI]** | | | **Low risk relapse [95%CI]** | | |
| --- | --- | --- | --- | --- | --- | --- | --- | --- | --- |
| **Treatment** | RT | ADT | RT+ADT | RT | ADT | RT + ADT | RT | ADT | RT + ADT |
| **All** | 32  (29-35) | 29  (26-32) | 16  (15-18) | 53  (48-59) | 8.0  (7.6-9.0) | 7.9  (7.2-8.6) | 41  (37-47) | 1.0  (0.9-1.3) | 2.6  (2.2-3.0) |
| **Life expectancy**  **< 15 years** | 24  (20-30) | 40  (33-50) | 13  (11-16) | 41  (35-48) | 12  (10-14) | 5.9  (5.0-6.9) | 30  (25-36) | 0.5  (0.4-0.8) | 2.7  (2.2-3.5) |
| **Life expectancy**  **≥ 15 years** | 33  (30-37) | 26  (23-29) | 17  (15-19) | 60  (53-69) | 6.2  (5.5-6.9) | 8.9  (8-10) | 50  (42-57) | 1.3  (1.1-1.7) | 2.5  (2.1-3.1) |
